# Intestinal permeability disturbances: causes, diseases and therapy

**DOI:** 10.1007/s10238-024-01496-9

**Published:** 2024-09-28

**Authors:** Barbara Macura, Aneta Kiecka, Marian Szczepanik

**Affiliations:** Faculty of Health Sciences, Institute of Physiotherapy, Chair of Biomedical Sciences, Jagiellonian University Medical College, Kopernika 7a, 31-034 Kraków, Poland

**Keywords:** Intestinal barrier, Gut permeability, Gut dysbiosis, Zonulin, Leaky gut diseases

## Abstract

Nowadays, a pathological increase in the permeability of the intestinal barrier (the so-called leaky gut) is increasingly being diagnosed. This condition can be caused by various factors, mainly from the external environment. Damage to the intestinal barrier entails a number of adverse phenomena: dysbiosis, translocation of microorganisms deep into the intestinal tissue, immune response, development of chronic inflammation. These phenomena can ultimately lead to a vicious cycle that promotes the development of inflammation and further damage to the barrier. Activated immune cells in mucosal tissues with broken barriers can migrate to other organs and negatively affect their functioning. Damaged intestinal barrier can facilitate the development of local diseases such as irritable bowel disease, inflammatory bowel disease or celiac disease, but also the development of systemic inflammatory diseases such as rheumatoid arthritis, ankylosing spondylitis, hepatitis, and lupus erythematosus, neurodegenerative or psychiatric conditions, or metabolic diseases such as diabetes or obesity. However, it must be emphasized that the causal links between a leaky gut barrier and the onset of certain diseases often remain unclear and require in-depth research. In light of recent research, it becomes crucial to prevent damage to the intestinal barrier, as well as to develop therapies for the barrier when it is damaged. This paper presents the current state of knowledge on the causes, health consequences and attempts to treat excessive permeability of the intestinal barrier.

## Introduction

The incidence of certain allergic, autoimmune and metabolic diseases has been increasing in recent decades and has reached almost epidemic dimensions. There is a significant increase in the incidence of allergic and autoimmune diseases, such as asthma, atopic dermatitis, allergic rhinitis, chronic sinusitis, food allergies, celiac disease and inflammatory bowel disease. Systemic and metabolic conditions such as diabetes, obesity, multiple sclerosis, rheumatoid arthritis, lupus erythematosus, ankylosing spondylitis, as well as Alzheimer’s disease, Parkinson’s disease, chronic depression and autism spectrum disorders also become a growing health problem [1–6].

One of various possible hypotheses for the rapid increase in the incidence of such conditions is the damage to the intestinal and/or respiratory epithelial/ skin integrity observed in their course and the associated disruption of the epithelial barrier, causing it to leak and increase permeability. Conditions such as asthma and atopic dermatitis are characterized by systemic type 2 immune responses. The migration of activated T lymphocytes, where they cause disease, has been shown for food allergen-specific and skin-homing T lymphocytes that are primed in the inflamed gut and migrate to the skin to cause atopic dermatitis. The mucosal barrier is crucial to protect the body from exogenous harmful biological and chemical agents, such as microorganisms and environmental pollutants. As such, what could have caused the weakening of the epithelial barrier in recent decades? Is it possible that, as a result of industrial development and environmental pollution, damage to the epithelial barrier has become so widespread that it has become one of the factors in increased incidence of so many diseases? Or is this phenomenon more of an effect of already developing disease than a cause? [1]. This paper presents the current state of knowledge on the role of intestinal barrier in the development of some diseases and attempts to treat them by repairing the epithelial barrier. A literature review was performed using the PubMed and Google School databases. We used the following descriptors and their combinations in our research: “intestinal/gut barrier/permeability”, “leaky gut/intestine”, “zonulin”, “gut dysbiosis”, “disease”, “cause”, “diagnosis”, “treatment”, “therapy”, “physical activity”, “exercises”. Only articles in English were selected and these fit the best to our research area.

## Intestinal barrier

The intestinal epithelium is the largest contact site between the external environment and the internal milieu. The function of gastrointestinal epithelial barrier is to protect against the entry of foreign antigens and microorganisms, while allowing the absorption of essential nutrients, water and electrolytes [2, 7].

The intestinal barrier consists of four components: microbial barrier, biochemical barrier, physical barrier and immune barrier [8].

The microbial barrier is the intestinal microbiota, located in the lumen of an intestine. The microbiota produces many metabolically active compounds that show antimicrobial activity and affect the function of the entire intestinal barrier. Commensal bacteria digest certain food components and also compete with pathogens for nutrients [8–11].

The biochemical barrier is mucus, which contains about 98% water [8] and, among others, mucins, glycoproteins, IgA antibodies, antimicrobial substances, produced by microorganisms—bacteria, viruses, fungi and intestinal cells. Mucus coats epithelial cells and protects them from the harmful effects of pathogenic microorganisms and toxic substances [8, 9, 12, 13].

The physical (epithelial) barrier is an essential component of the entire intestinal barrier. It consists of a single layer of specialized cells: enterocytes, goblet cells (produce mucins), Paneth cells (produce antimicrobial peptides and proteins), enteroendocrine cells, M cells and intestinal stem cells. These cells undergo renewal every 3–5 days. Epithelial cells have a variety of functions and are closely interconnected [8, 9, 14, 15].

The immune barrier is associated with the presence of lymphoid tissue in the intestines known as gut-associated lymphoid tissue (GALT). The GALT system is located in the mucosa and submucosa of the intestines, directly beneath the epithelial cells. This system consists of intraepithelial lymphocytes (IELs), Peyer’s patches, which are clustered lymphoid papules, and lymphocyte clusters. The GALT system has also been found to contain antigen-presenting cells (APCs), T lymphocytes, B lymphocytes, plasma cells, as well as macrophages, mast cells and granulocytes. The secretory IgA antibody (sIgA) is synthesized in the intestine in particular [8, 9, 16, 17].

**Fig. 1:**
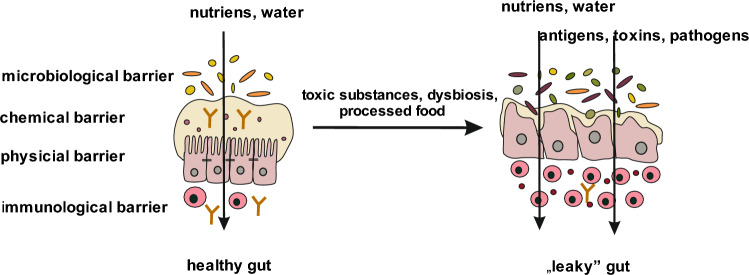
Gut barrier. The intestinal barrier consists of 4 layers: microbiological, chemical, physical and immunological. Under the influence of exogenous or endogenous factors, the intestinal barrier is destroyed. This phenomenon is called "leaky gut"

Most dietary proteins undergo endocytosis by intestinal epithelial cells. Lysosomal degradation leads to the breakdown of proteins into smaller peptides, thus avoiding activation of the immune system. Fluids and solutes are transported between cells. This transport is regulated by tight junctions (TJ) [18]. The intestinal barrier is a selective barrier—its function is to allow the transport of digested food essential for the body’s function, but at the same time to keep harmful substances and microorganisms in the intestinal lumen, which requires strict regulation of the barrier’s permeability [9, 19]. The TJ between enterocytes play a key role in providing an intestinal barrier. These junctions are composed of proteins, including occludin, claudin and junctional adhesion molecules (JAMs). TJ-building proteins bind to actin proteins, which are part of the cell’s cytoskeleton, via special zonula occludens proteins (ZO). These proteins are the target element for molecules that regulate TJ function, and are therefore responsible for the regulation of permeability of the intestinal barrier [18, 19] (Fig. 1).

### Factors that can damage the intestinal barrier

The external environment, as a result of urbanization and globalization, has changed significantly in recent decades. Pollution and climate change, chemical compounds commonly used in industry and households, ecosystem changes, unhealthy diet, and stimulants, mainly alcohol, tobacco and e-cigarettes, may disrupt the epithelial barriers of the skin and mucosal surfaces. Air, water and food pollution, microplastic particles, nanoparticles, household chemicals and tobacco smoke are the most common epithelial barrier disrupting factors. Complex interactions between all these factors affect organisms, the end result of which depends on the combination of these factors and the body’s susceptibility [2].

It is well known that in recent decades the contamination with chemicals of the food we eat and the water we drink has increased significantly, if only by widely used food additives, pesticides or additives added to livestock feed. Recently, there has been increasing reports of food contamination with microplastics and nanoparticles. Such substances can easily penetrate tissues and interact with cellular structures [2, 20, 21]. Microplastics cause changes in protein structure [2], interact with cell membrane lipids, induce transcription of inflammatory genes and increase production of pro-inflammatory cytokines, lead to dysfunction of the endoplasmic reticulum, mitochondria and induce cell death due to oxidative stress. Nanoparticles also cause changes in protein structure and interact with cell membrane lipids [2, 22, 23]. Nanoplastics have been shown to induce transcription of inflammatory genes, increase levels of pro-inflammatory cytokines, and alter the expression of certain proteins [2, 24]. Microplastics have a high rate of absorption in the gastrointestinal tract and accumulate in the external environment and living organisms [2, 21]. Experiments on mice have revealed that polystyrene microplastics damage the intestinal barrier and reduce intestinal mucus production [2, 25]. Table 1 shows the main factors considered to have a deleterious effect on the intestinal barrier status: microplastic [20–25], nanoparticles [26–29], detergents and emulsifiers [30–36], the state of the intestinal microbiota [37–45] and diet [46–51].

**Table 1:** The main factors damaging the intestinal barrier include environmental factors, microbiological factors and dietary factors. Each of these groups exerts broad and diverse biological effects on the state of intestinal barrier

Factor	Definition	Biological effects
*Environmental substances*		
Microplastic	Plastic waste with a diameter of less than 5 mm can be an environmental pollutant or accumulate in living organisms	Interaction with cell structures, cell membranes, proteins and changes in their function increased gene expression of pro-inflammatory and pro-apoptotic factors induction of apoptosis [20–25]
Nanoparticles	A fragment of matter with a dimension not exceeding 100 nm (nm) can be formed naturally in the environment or used in industry, such as cosmetics	Stimulation of collagen production and its deposition in the extracellular matrix, which can lead to fibrosis destabilization of mitochondrial and lysosome function disruption of the integrity of phospholipid and lysosomal membranes disruption of intercellular connections and increase in cell permeability induction of apoptosis [26–29]
Detergents and emulsifiers	Detergents and emulsifiers are surfactants detergents are used for cleaning, emulsifiers are mainly used in the food industry	TJ damage increased production of certain interleukins changes in microbiota composition, disruption of mucus-bacteria interactions in the intestines [30–36]
*Status of intestinal microbiota*		
Medications (especially antibiotics), stress, low physical activity, alcohol, Dietary changes (low fiber, meals high in simple sugars)	Dysbiosis—an imbalance of microbiology, involving a change in the composition of normal microbiota	Loss of biodiversity decrease in the number of commensals increase in opportunistic pathogens [37–45]
*Diet*		
Unhealthy diet	Processed food—preservatives, emulsifiers, artificial colors, enzymes, surfactants, high amount of saturated fatty acids, incorrect amount and ratio of omega-3 and omega-6 fatty acids	Increase in the incidence of food allergies weakening of the intestinal barrier [46–51]

### Dysbiosis vs intestinal tightness

Exposure to environmental factors can directly weaken the integrity of the intestinal epithelial barrier and alter the microbiome structure. “Leaky” epithelium and intestinal dysbiosis, often concomitant phenomena, cause the development of inflammation. This is also due to the direct proximity of immune cells, located under the layer of intestinal epithelial cells [1, 2]. Under the influence of a leaky epithelial barrier, the immune system is locally activated and inflammation develops, which can be transmitted via immune cells and the cytokines they secrete to other systems and organs. This is the reason why diseases, the development of which, at least in part, attempts to link to a leaky epithelial barrier, can have such different localizations [1]. Often after damage to the intestinal epithelium, colonization with opportunistic pathogens occurs, and commensal abundance and biodiversity begins to decline [40, 41, 43–45, 52]. Translocation of microorganisms into the intercellular compartments and further into the deeper tissue layers causes microinflammation. Exposure to various chemical compounds can also induce epigenetic changes that modulate the immune system and result in the facilitated development of certain diseases [1, 2, 37].

It is widely accepted that the microbiome plays a key role in the intestinal epithelial well-being. As mentioned above, a normal microbiota regulates various aspects of epithelial barrier function, such as TJ expression, angiogenesis, vascular permeability and immunomodulation [2, 37]. In the case of a leaky epithelial barrier, commensals and opportunistic pathogens migrate below the epithelial cell layer. This results in the development of inflammation, which is associated with the development of certain diseases. It has been postulated that the increase in allergic diseases may be due to bacterial dysbiosis and reduced biodiversity of the healthy microbiota [1, 2, 39].

In turn, the state of intestinal microbiota is influenced by a wide range of factors, with the main ones we can include diet, medications used, especially antibiotics, psychotropic drugs, proton pump inhibitors (PPIs) and genetic predisposition. There are speculations that non-pathogenic commensal microorganisms, which co-evolved with humans, have been a source of immunomodulatory signals for the human body (the “old friends” hypothesis), and now prevent the development of immune-mediated chronic diseases (the “hygiene hypothesis”), with the proper composition of the microbiome promoting the maintenance of the body’s immune balance (the “biodiversity hypothesis”) [1, 39, 53, 54]. In general, these hypotheses present the assumption that microbiota of proper composition regulates epithelial barrier function, including by regulating barrier permeability and TJ, influencing vascular permeability and angiogenesis, and affecting local inflammation and GALT tolerance [1, 55, 56]. Dysbiosis, through the lack of appropriate immunoregulatory and barrier factors, such as, e.g., short-chain fatty acids, causes inappropriate activation of the immune system, resulting in epithelial inflammation, epithelial barrier damage and disease development [55, 57–61]. This activation is characterized by a predominance of T helper 2 (TH2) lymphocytes, innate lymphoid cells type 2 (ILC2) and eosinophils. Mast cells, macrophages and lymphocytes may also be involved in this reaction. A vicious cycle emerges: dysbiosis—epithelial damage—chronic inflammation, which results in the epithelium not regenerating [1].

Inflammation, smoldering in the damaged intestinal epithelium, activates immune cells that can migrate and cause inflammation to develop in other organs [62, 63]. The relationship between disruption of the epithelial barrier in the intestine and the development of diseases in other organs is now widely studied [1] (Fig. 1).

The detailed interrelationships and interdependencies between substances that damage the epithelial barrier, the intestinal microbiota, the immune system, the development of inflammation, changes in cell function and ultimately the development of diseases, often located distantly from the intestine, remain unclear.

### Role of zonulin in the regulation of TJ function

Zonulin is a paracrine protein with a molecular weight of 47 kDa. This protein is produced by several cells in the body, including epithelial cells of the small intestine. Zonulin has the ability to reversibly regulate the function of TJs [9, 16, 18, 64–66]. It is likely that zonulin release is initiated by dysbiosis and gliadin by a similar mechanism. On enterocytes and monocytes, gliadin binds to the CXCR3 chemokine receptor, which provides a signal for the cell to increase zonulin synthesis. Zonulin is then secreted into the intestinal lumen activates the epidermal growth factor receptor (EGFR) via protease-activated receptor 2 (PAR2). The activation of an intracellular cascade of biochemical reactions results in the phosphorylation of zona occludens (ZO) protein and myosin, as well as actin polymerization. These processes result in the detachment of, among others, the ZO protein from the TJ complex and impair integrity of the TJ [16, 18, 66–69].

Increased zonulin production has been observed under the presence of certain bacteria and food components, such as gluten. Excessive release of zonulin results in weakening of TJ and consequent passage of antigens into the vicinity of immune cells and into the circulatory system. As a result, local inflammation develops, and activated immune cells and cytokines can affect other organs or trigger immune-related diseases, e.g., autoimmunity. Excessive secretion of zonulin causes prolongation of the opening time of TJ, which can prolong the abnormal activation of the immune system over time [16, 18, 70–72]. Elevated zonulin levels have been found in patients with rheumatoid arthritis, multiple sclerosis or ankylosing spondylitis [1, 73–75].

**Fig. 2:**
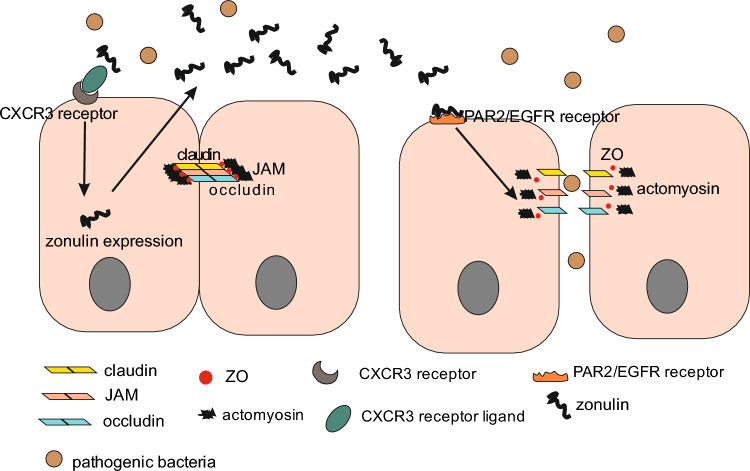
Mechanism of zonulin secretion. In the course of inflammation, CXCR3 receptors are activated, leading to the expression of zonulin protein in intestinal epithelial cells. Zonulin is secreted into the extracellular space and then activates PAR2/EGFR receptors, which triggers a biochemical cascade inside the cell and ultimately causes TJ relaxation

There are reports that indicate that the increase in intestinal permeability in response to bacterial exposure is abolished by administration of a synthetic inhibitor of peptide binding to the zonulin receptor [18]. The release of zonulin is a physiological mechanism that regulates microbial colonization in the small intestine [18, 66, 76]. Zonulin transgenic mice showed resistance to “normalization” of the microbiota by transferring the normal microbiota. Therefore, a dysregulated zonulin system may also be the cause of intestinal dysbiosis [68, 77] (Fig. 2).

### Relationship between disrupted intestinal barrier and disease incidence

Three basic elements are interdependent: altered microbiota, disrupted intestinal barrier, disease. However, the directionality of this relationship is controversial. Is it the abnormal intestinal microbiota that causes increased intestinal permeability and translocation of bacteria as well as their products and the development of disease, or is it the disease that causes a systemic inflammatory response resulting in disruption of the intestinal barrier, subsequent translocation of bacteria and further damage to the organ? Therefore, is leaky gut a cause or an effect of disease? [17, 78–80]. There are also reports that rupture of the respiratory or intestinal epithelial barrier and weakening of other barriers, for example, the blood–brain barrier or the vascular endothelial barrier, may facilitate the development of metabolic and autoimmune diseases [55, 81–84].

For example, a growing number of studies indicate that dysbiosis may promote the development of hypertension, and hypertension may affect the composition of the intestinal microbiota. Proper microbiota status is a factor that promotes normalization of blood pressure, and proper blood pressure promotes normalization of microbiota status. A correlation is apparent between these factors, but it is difficult to clearly determine its direction. Results indicate that the prevalence of intestinal barrier dysfunction is higher in patients with hypertension than in those without hypertension [45, 85–88]. Perhaps intestinal barrier damage may be linked to the development of hypertension [89–91]. Impaired barrier function does not simply and directly lead to disease in animal models of disease. It is also uncertain whether improvement of barrier function can, and to what extent, alter the course of disease [92–94]. A dysfunctional intestinal barrier can thus have many possible causes and produce symptoms that can range from gastrointestinal disorders such as bloating, cramping, and food intolerance to inflammatory bowel diseases (IBD), systemic, neurological, and psychiatric diseases [17].

Of particular interest are the findings on the link between the leaky gut barrier and disease incidence in humans. Inflammatory bowel diseases, which include ulcerative colitis and Crohn’s disease, are chronic diseases with an incompletely understood etiology [95, 96]. Intestinal leakage in IBD patients is associated with dysbiosis, inflammatory response and TJ abnormalities. The intestinal microbiota of IBD patients is characterized by an increase in pro-inflammatory bacteria and altered expression of multiple cytokines [95, 97, 98]. Altered intestinal permeability has been shown to occur in asymptomatic patients before the onset of clinical symptoms [95, 99, 100].

The intestines and liver are anatomically and functionally interconnected, forming the so-called gut liver axis [101–105]. Increased dysbiosis, impaired intestinal barrier, bacterial translocation and pro-inflammatory response play an important role in the development of chronic liver disease. Inflammation worsens liver damage and promotes the development of cirrhosis and its complications [79, 80, 103, 104]. Research data indicate that damage to both the intestinal epithelium and vascular barrier is necessary for the development of non-alcoholic steatohepatitis, which increases the role of intestinal dysbiosis in the process. Alcoholic liver disease is also associated with intestinal dysbiosis and disruption of the intestinal barrier, as both alcohol and its metabolites are toxic agents for intestinal cells. Impaired not only the intestinal epithelial barrier, but also the vascular barrier is responsible for damage to intestinal epithelial function in alcoholic liver disease [106–108].

Elevated serum zonulin levels, as well as bacterial dysbiosis and a leaky gut barrier, have been observed in patients with rheumatoid arthritis, while subclinical intestinal inflammation has been observed in patients with ankylosing spondylitis [74, 75]. Activation of Paneth cells in response to intestinal dysbiosis may be responsible for early symptoms of this disease, confirming the existence of an intestinal-articular axis. It has been observed that patients affected by systemic lupus erythematosus are colonized by a more homogeneous intestinal microbiota [1, 74, 75, 95].

In Parkinson’s disease, patients complain of gastrointestinal symptoms years prior to diagnosis [95, 109]. α-Synuclein, a protein typical of Parkinson’s disease, is synthesized in the intestines and then carried to the central nervous system via the vagus nerve [95, 110, 111]. In contrast, children with autism spectrum disorders develop intestinal symptoms, such as constipation, abdominal pain and diarrhea [95, 112]. Studies on the intestinal microbiota have shown an inverse relationship between microbiota diversity and neurological disorders, a reduction in *Bacteroidetes*, and reduced expression of TJ proteins in the intestinal mucosa [95, 113–115].

Other studies have shown that changes in intestinal permeability occur before the onset of type 1 diabetes symptoms, and a change in TJ and dysbiosis associated specifically with a decrease in butyrate-producing bacteria have also been observed [1, 95]. Changes in the composition of intestinal microbiota in patients with type 2 diabetes worsen the abnormal functioning of intestinal barrier and increase the pro-inflammatory state. This leads to increased insulin resistance and impaired beta cell function [1, 95, 116]. Moreover, in patients with type 2 diabetes, hyperglycemia promotes the maintenance of pro-inflammatory state [95, 117, 118]. Obesity, in turn, is associated with chronic low-grade inflammation. It is associated with pro-inflammatory macrophage activity in adipose tissue. Dysbiosis is also more common in obesity [95, 119] (Table 2).

**Table 2:** Diseases postulated to have a leaky intestinal barrier, their etiology, part of the body affected by the disease and possible therapy for the permeable intestinal barrier, mainly in relation to IBD

Disease [95, 120]	Proposed etiology-often multifactorial and/ or unknown [95, 120]	Part of the body affected by the disease [95, 120]	Possible therapy for the permeable intestinal barrier, mainly in relation to IBD [121–123]
*Immune diseases*			Pharmacological treatments: *typical drug therapy:* aminosalicylates, corticosteroids (CS), immunomodulators, and biologics drugs, (anti-TNF therapy, anti-IL-12/23 therapy, anti-integrin therapy) *other medicines:* robiotics, prebiotics glutamine vitamin D, A metformin Non-pharmacological treatments: healthy diet medical herbs phenolic compounds physical activity fecal microbiota transplantation (FMT) More information about these compounds is included in section “Possibilities for diagnosis and regeneration of damaged intestinal barrier”
Inflammatory bowel diseases (IBD), such as ulcerative colitis (UC) and Crohn’s disease (CD)	Multifactorial; also immunological	Intestine	
Irritable bowel syndrome	Multifactorial; also immunological	Intestine	
Celiac disease	Factors: mainly genetic, autoimmunological	Intestine	
Rheumatoid arthritis	Multifactorial; autoimmunological	Joints	
Ankylosing spondylitis	Multifactorial; autoimmunological	Spine joints	
Systemic lupus erythematosus	Multifactorial; autoimmunological	Various tissues	
Type 1 diabetes	Autoimmunological	Damage to pancreatic beta cells	
*Liver diseases*			
Liver cirrhosis	Alcohol, hepatitis B, C or D	Liver	
Non-alcoholic fatty liver disease (NAFLD)	Unhealthy diet, obesity, lack of physical activity	Liver	
Alcoholic liver disease (ALD)	Alcohol	Liver	
*Metabolic diseases*			
Type 2 diabetes	Unhealthy diet, obesity, lack of physical activity	Tissue insulin resistance	
Obesity	Unhealthy diet, lack of physical activity	Adipose tissue	
*Neurological diseases*			
Alzheimer’s disease	Multifactorial	Brain	
Parkinson’s disease	Unknown	Brain	
Major depression disorder	Factors: genetic, psychological stress	Brain	
Autism spectrum disorders	Multifactorial	Brain	

### Possibilities for diagnosis and regeneration of damaged intestinal barrier

Evaluation of the level of intestinal barrier permeability is possible during indirect or direct diagnostic tests [124].

Indirect tests involve oral administration of test substances, followed by measurement of their concentration in blood or urine. The most commonly administered substance in indirect tests are sugars. The lactulose/mannitol test (L/M test), the most popular sugar test, assesses small intestinal permeability by measuring the excretion of these substances in the urine. Lactulose is a large oligosaccharide that is adsorbed only when the intercellular junctions leak; mannitol is a smaller molecule that can freely penetrate the intestinal barrier. The L/M test is non-invasive and has high sensitivity. It is also possible to administer other sugars and multi-sugar tests [124].

In addition, there are also possibilities to measure the level of substances of endogenous origin in the blood. Such biomarkers include zonulin, fatty acid binding proteins (FABP), citrulline, glucagon-like peptide (GLP)-2, LPS, LPS-binding protein (LBP) or fecal α1 antitrypsin (AAT), for example. However, these tests are not as sensitive as sugar tests [120, 125].

New imaging techniques, particularly confocal laser endomicroscopy, allow in vivo evaluation of the integrity of intestinal barrier after intravenous administration of fluorescein as a contrast agent. Fluorescein does not reveal paracellular transport. Confocal laser endomicroscopy is currently used in the diagnosis of gastrointestinal cancers, as well as in irritable bowel syndrome or celiac disease. Magnification of up to a thousand times allows detection of pathological changes within the intestinal epithelium [124].

Conventional treatments for IBS alleviate disease symptoms with pharmacotherapy. The most commonly used drugs are aminosalicylates, corticosteroids (CS), immunomodulators (for example, thiopurines (TPs), methotrexate (MTX), calcineurin inhibitors, Janus kinase (JAK) inhibitors) and biologics drugs. Biological therapies mainly include inhibitors of pro-inflammatory cytokines and integrin antagonists: anti-TNF therapy, anti-IL-12/23 therapy, anti-integrin therapy. Biological therapies, especially anti-TNF therapy are effective treatments, but the primary lack of response to TNF inhibitors or the secondary loss of response among some patients requires the search for new therapeutic solutions [121–123]. Importantly, the inflammatory environment may negatively affect the function of epithelial stem cells, and thus the reconstitution of healthy epithelium may be hindered [126–129]. Leaky gut treatment should begin with an attempt to determine the causes of intestinal barrier damage and then eliminate this factor. Until the intestinal barrier is restored, it seems reasonable to use drugs that alleviate inflammation in the intestine.

One method of restoring normal intestinal microbiota is fecal microbiota transplantation (FMT). This therapy involves the transfer of gastrointestinal microbiota obtained from a healthy donor to the gastrointestinal tract of a recipient, i.e., a person with known dysbiosis [86].

The fermentable oligosaccharides, disaccharides, monosaccharides and polyols (FODMAP) diet is a diet based on short-chain carbohydrates and polyols. These compounds are poorly absorbed and rapidly ferment, and due to their osmotic properties cause increased water content in the intestinal lumen [130–132]. Consumption of FODMAPs has broad effects on the digestive system. A FODMAP diet has a beneficial effect on the intestinal microbiota composition and function. And as we know, proper microbiota helps maintain the tightness of the intestinal barrier. However, patients with irritable bowel syndrome or IBD are better off with a low-FODMAP diet, as it can exacerbate disease symptoms due to gas production and stretching of the intestinal lumen [8, 130–132].

Probiotics are live microorganisms that have beneficial effects on the host’s health. Probiotic administration reduces intestinal leakage by, among others, affecting intestinal immunoregulation and anti-inflammatory effects (for example, an increase in sIgA production), anti-inflammatory effects, strengthening the epithelial barrier (for example, an increase in the synthesis of mucin and short-chain fatty acids (SCFAs), and the production of bacteriocins, which limit the growth of pathogenic microorganisms (for example, β defensin) [8, 133, 134]. Probiotics also increase the synthesis of proteins that constitute the TJ. The main probiotics showing protective effects on the state of the intestinal barrier include *Lactobacillus rhamnosus* GG*, Lactobacillus acidophilus, Lactobacillus plantarum, Bifidobacterim infantis, E. coli Nissle* 1917 *and Bifidobacterium animalis lactis* BB-12 [8, 133–136].

Research results indicate that vitamins A and D affect the intestinal barrier in an indirect and multidirectional manner [137, 138]. In humans, these vitamins increased the diversity of microbiota compared to their deficiency [8, 139], improved TJ [8, 140, 141], inhibited the production of IFN-γ by T cells [8, 142] and inhibited Th17 cells and promoted the synthesis of IL-10 and FOXP3 protein [8, 143]. Retinoic acid stimulates the synthesis of antimicrobial factors [8, 144]. However, there is a small number of studies on this issue [8].

Another substance that has a positive effect on the state of intestinal barrier is dietary fiber. Fermentation of dietary fiber in the gastrointestinal tract produces SCFAs, such as butyrate, propionate and acetate. Fermentation occurs with the participation of beneficial bacteria, mainly *Lactobacillus* and *Bifidobacterium* [8, 145]. Research results indicate that butyrate affects mucin levels and TJ status. Short-chain fatty acids affect immune cell function, leading to changes in the levels of released cytokines and reactive oxygen species. Deficiency of fiber and short-chain acids can lead to increased intestinal permeability [8, 145–147].

Glutamine is considered a key amino acid capable of regulating the expression of TJ proteins. Findings indicate that glutamine has anti-inflammatory effects and reduces intestinal mucosal permeability, especially when combined with probiotics. However, the results of preliminary studies indicate that detailed studies are needed [148, 149]. Similarly, the suggested beneficial effect of arginine on the state of intestinal barrier requires further research. The beneficial effects of polyphenols on the state of intestinal epithelium are associated with beneficial effects on the state of TJs and an increase in mucus production, as well as effects on the activity of several protein kinases. Polyphenols also increase the activity of antioxidant enzymes. Research results suggest that a diet rich in polyphenols reduces the risk of intestinal barrier dysfunction [8, 150].

Medicinal herbs are used to treat autoimmune diseases associated with leaky gut, such as ulcerative colitis [8, 151]. The mechanism of medicinal plants is broad, and includes regulation of intestinal microbiota composition and intestinal permeability, increased expression of both mRNA and claudin-1 protein, and anti-inflammatory effects (Chinese tea, hibiscus, liquorice, marshmallow, ginger root, peppermint, plantain, curcumin). The mechanisms of action and efficacy of herbs remain inconclusive [8, 151–153].

Mushrooms are a source of bioactive compounds, such as vitamin D and phenolic compounds and also a source of prebiotics, as they contain various polysaccharides. Thus, they modulate the intestinal microbiota by stimulating the production of catecholamines and their metabolites as well as affecting the inflammatory response [8, 154, 155].

It is also well known that physical activity can increase the diversity of intestinal microbiota, enhance SCFA production and stimulate anti-inflammatory mechanisms, mainly aerobic exercises, such as running, cycling, fitness exercises [86, 156, 157].

Research results indicate that physical activity regulates the level of intestinal barrier permeability in a manner that is dependent on exercise intensity. Regular, moderate-intensity physical exercise has a positive effect on intestinal epithelial status and intestinal barrier integrity. Exercise increases the diversity of intestinal microbiome, which, as mentioned above, contributes to maintaining the integrity of the intestinal barrier, and also shows anti-inflammatory and antioxidant effects. Moderate physical activity can be an adjunctive factor in the treatment of leaky gut [158, 159]. High-intensity physical activity, typical of competitive or extreme sports, is correlated with a high incidence of gastrointestinal disorders and symptoms of increased intestinal permeability. This is a stressor for the body, causing hypoxia of intestinal cells due to redistribution of blood in the body, hyperthermia and dehydration [158, 159].

Therapy for leaky gut syndrome should include dietary modification and supplementation with probiotics and prebiotics and, perhaps the aforementioned compounds. Treatment of minor disorders can be achieved with a non-pharmacological dietary approach. Treatment with a zonulin antagonist inhibited the development of arthritis in mouse models of rheumatoid arthritis [1, 17]. Epigenetic changes may also underlie epithelial barrier leakage. Some experiments have shown that inhibition of histone deacetylase can restore barrier tightness [128, 160]. This issue still requires further research.

## Conclusion

Contemporary recommendations regarding the described risks associated with a leaky gut barrier include, first and foremost, the prevention of epithelial barrier damage. This can be done by beginning with examining substances for which there are reports of their potential toxicity to the intestinal barrier and determining the degree of toxicity, monitoring the concentrations in the environment and various products of compounds toxic to the intestinal barrier, as well as limiting contact with substances that may cause intestinal leakage to doses that could be considered safe. An alternative is to develop substances that are safer for the epithelial barrier and are substitutes for commonly used, dangerous compounds [1].

The relationship between barrier function and clinical symptoms of diseases is often unclear and anecdotal, and this issue requires particularly thorough research. However, regardless of the degree of involvement of the leaky gut barrier in the development of certain diseases, the persistent inflammation that accompanies damage to the intestinal barrier negatively affects the functioning of gastrointestinal tract and the whole body. It is necessary to continue molecular research on the epithelial barrier in order to develop preventive guidelines for maintaining its tightness, to develop new methods for evaluation of its functionality and therapy in case of diagnosed damage.

The study on the relationship between the intestinal microbiota and certain diseases may help to understand the broader etiology of these conditions, including the influence of microbiota on their development. Understanding these mechanisms is very important in the prevention and treatment of many diseases. Combining traditional forms of treatment, mainly pharmacotherapy for conditions, with normalization of the intestinal barrier and the state of microbiota can greatly enhance the efficacy and effectiveness of many therapies.

## Data Availability

No datasets were generated or analyzed during the current study.
